# 5-Hydroxytryptamine Modulates Maturation and Mitochondria Function of Human Oligodendrocyte Progenitor M03-13 Cells

**DOI:** 10.3390/ijms22052621

**Published:** 2021-03-05

**Authors:** Simona Damiano, Giuliana La Rosa, Concetta Sozio, Gina Cavaliere, Giovanna Trinchese, Maddalena Raia, Roberto Paternò, Maria Pina Mollica, Vittorio Enrico Avvedimento, Mariarosaria Santillo

**Affiliations:** 1 Dipartimento di Medicina Clinica e Chirurgia, Università di Napoli “Federico II”, 80131 Napoli, Italy; giuli_la-ro@libero.it (G.L.R.); sozioimma@gmail.com (C.S.); rpaterno@unina.it (R.P.); 2 Dipartimento di Biologia, Università di Napoli “Federico II”, 80126 Napoli, Italy; gina.cavaliere@unina.it (G.C.); giovanna.trinchese@unina.it (G.T.); mariapina.mollica@unina.it (M.P.M.); 3 Dipartimento di Medicina Molecolare e Biotecnologie Mediche, Università di Napoli “Federico II”, 80131 Napoli, Italy; raia@ceinge.unina.it (M.R.); avvedim@unina.it (V.E.A.)

**Keywords:** 5-hydroxytryptamine, oligodendrocyte precursor cells, reactive oxygen species, NOX, migration, proliferation, differentiation, mitochondria

## Abstract

Inside the adult CNS, oligodendrocyte progenitor cells (OPC_S_) are able to proliferate, migrate and differentiate into mature oligodendrocytes (OLs) which are responsible for the production of myelin sheet and energy supply for neurons. Moreover, in demyelinating diseases, OPCs are recruited to the lesion areas where they undergo differentiation and myelin synthesis. Serotonin (5-hydroxytryptamine, 5-HT) is involved in OLs’ development and myelination, but so far the molecular mechanisms involved or the effects of 5-HT on mitochondria function have not yet been well documented. Our data show that 5-HT inhibits migration and proliferation committing cells toward differentiation in an immortalized human oligodendrocyte precursor cell line, M03-13. Migration blockage is mediated by reactive oxygen species (ROS) generation since antioxidants, such as Vit C and Cu-Zn superoxide dismutase, prevent the inhibitory effects of 5-HT on cell migration. 5-HT inhibits OPC migration and proliferation and increases OL phenotypic markers myelin basic protein (MBP) and Olig-2 via protein kinase C (PKC) activation since the inhibitor of PKC, bis-indolyl-maleimide (BIM), counteracts 5-HT effects. NOX inhibitors as well, reverse the effects of 5-HT, indicating that 5-HT influences the maturation process of OPCs by NOX-dependent ROS production. Finally, 5-HT increases mitochondria function and antioxidant activity. The identification of the molecular mechanisms underlying the effects of 5-HT on maturation and energy metabolism of OPCs could pave the way for the development of new treatments for autoimmune demyelinating diseases such as Multiple Sclerosis where oligodendrocytes are the primary target of immune attack.

## 1. Introduction

Oligodendrocyte (OLs) are glial cells of the CNS involved in the formation of myelin sheath around neuron axons. Myelination assures the fast saltatory transmission of the action potentials and energy saving for neurons. However, OL functions are not restricted to myelination but they also provide metabolic support to neurons [1]. OLs produce lactate, which can be shuttled through the lactate transporters MCT1, MCT2 and MCT4 [2] to axons to generate metabolic energy in the form of ATP [3]. An emerging additional role of OL and myelination is that played in neuronal circuits’ plasticity as response to brain activity, a process known as adaptive myelination [4].

During CNS development, OL originates from Oligodendrocyte Progenitor Cells (OPC) deriving in turn from neural progenitor cells (NPCs) [5]. This process is regulated by different molecules such as growth factors, cytokines, hormones, and neurotransmitters [6]. Oligodendrocyte development must be adequate to the number of nerve fibers that must be myelinated; indeed, neuronal activity modulates the oligodendrocyte migration along the axons, proliferation and differentiation through growth factors and small neurotransmitter release acting on proper receptors expressed by oligodendrocytes [7,8,9,10].

In adulthood, OPCs are the major dividing cells of the CNS, representing 5% of the total cells. In the adult CNS, a large number of OPC retain the ability to migrate, proliferate and differentiate to myelinating OL [11], assuring a continuous turnover of mature OL and adaptive myelination [4]. Furthermore, a notable function of OPC is remyelination that take place at the level of white matter lesions in demyelinating diseases like multiple sclerosis (MS) where OLs are the primary cellular target of an immune attack [5].

The OPCs microenvironment plays a key role in the regulation of migration, proliferation and differentiation of these cells especially inside MS lesions. Increasing evidence suggest that OPC are sensitive to cytokines and also have immunomodulatory properties. Exposure of OPC or mature OL to INFgamma induces cell death [12] while TNFalpha induce proliferation and myelination [13]. Moreover, in human MS brain and in EAE mouse, an animal model of multiple sclerosis, disease-specific OL have been found; these cells, which are involved as well as OPCs in antigen processing and presentation [14,15], promote tissue damage and remyelination blocking. Therefore, in MS, OL are not only the target but also a determinant of autoimmune attack.

Reactive oxygen species (ROS) can induce cellular damage [16,17] or can function as mediators of intracellular signaling [18,19], depending on levels and the source of production. Adaptive antioxidant mechanisms involving antioxidant enzymes and various antioxidant molecules limit the increase in ROS [20,21,22].

OPC differentiation and remyelination are also modulated by reactive oxygen species (ROS). The main deliberated source of ROS are NOX enzymes, membrane NADPH oxidases producing superoxide anions by one electron reduction of oxygen using NAD(P)H as the electron donor [23]. Many membrane receptors relay on NOX-dependent ROS production for downstream signaling. As examples, NOX enzymes are activated by growth factor receptors such as platelet-derived growth factor receptor [24,25,26,27], epidermal growth factor receptor [28] cholinergic receptors [29] and many others [30,31]. NOXs are widely expressed in the central nervous system cells, including oligodendrocytes [32] where they mediate redox signaling leading to OPC differentiation [18].

Mitochondria are highly dynamics organelles that have a crucial role in the generation of ATP by oxidative phosphorylation, but additional functions include the generation of reactive oxygen species [33]. ROS are produced by mitochondria as a byproduct of oxidative metabolism through the one-electron reduction of molecular oxygen forming a superoxide anion [34]. The presence of functional mitochondria is likely necessary to maintain healthy oligodendrocytes that have high energy demands and are extremely vulnerable to energy deprivation [35]. Several pieces of evidence demonstrated that NO also affects oligodendrocytes’ energy metabolism by impairing the function of mitochondria [36]. Dysfunctional mitochondria can lead to excessive mitochondria-generated ROS that induce peroxidation of lipids, protein and DNA, resulting in axonal energy failure and subsequent neurodegeneration and the activation of apoptotic pathways [37].

Serotonin has a well-established regulatory role in neuronal differentiation and migration, axonal growth, synaptogenesis and circuit formation during brain development [38]. Some data link 5-hydroxytryptamine (5-HT) mediated signaling to OL development and functions. Manipulation of 5-HT levels affects normal OL functions; indeed, early life exposure to selective 5-HT reuptake inhibitors (SSRIs) in rats led to disruption of OL development and myelination and to the onset neurological symptoms and abnormal social behavior [39]. These in vivo data are also supported by experiments conducted in primary OL progenitor cells (OPCs) obtained from rat forebrain and on OL at different developmental stages obtained from OPCs cultured in differentiation medium showing that chronic exposure to 5-HT impairs OL development and myelination [40].

Despite the evidence that serotonin pathway disruption affects oligodendrocyte functions, the exact physiological role of the 5-HT receptors during oligodendrocyte maturation is unclear. Moreover, although it is known that in rodent cortical neurons 5-HT increases mitochondria biogenesis and function via the 5-HT2AR/SIRT-1/PGC1 alpha axis [41], no data are available about the effects of 5-HT on mitochondria function in oligodendrocytes.

To ascertain the stages of OL development affected by 5-HT and the molecular mechanisms involved, we used an immortalized oligodendrocyte cell line, the M03-13 cells. These cells have the biological feature of OPC, and they migrate, proliferate and, if properly stimulated, differentiate up to the stage of premyelinating OL. Our data show that 5-HT increases mitochondrial and NOX-dependent ROS levels negatively modulating migration and proliferation and favoring differentiation of oligodendrocyte cells through PLC/PKC/ROS-dependent pathways. In addition, 5-HT increases mitochondria oxidative capacity. These data, highlighting novel mechanisms of action of 5-HT in OLs biology, can be potentially useful for the development of new therapies capable of promoting remyelination in demyelinating diseases.

## 2. Results

### 2.1. 5-HT Inhibits M03-13 Cells Migration

**Figure 1:**
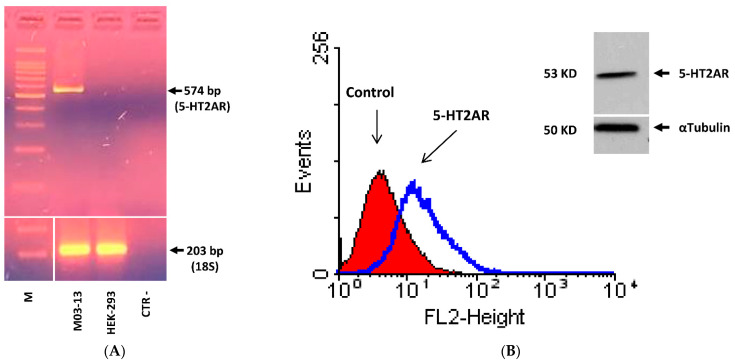

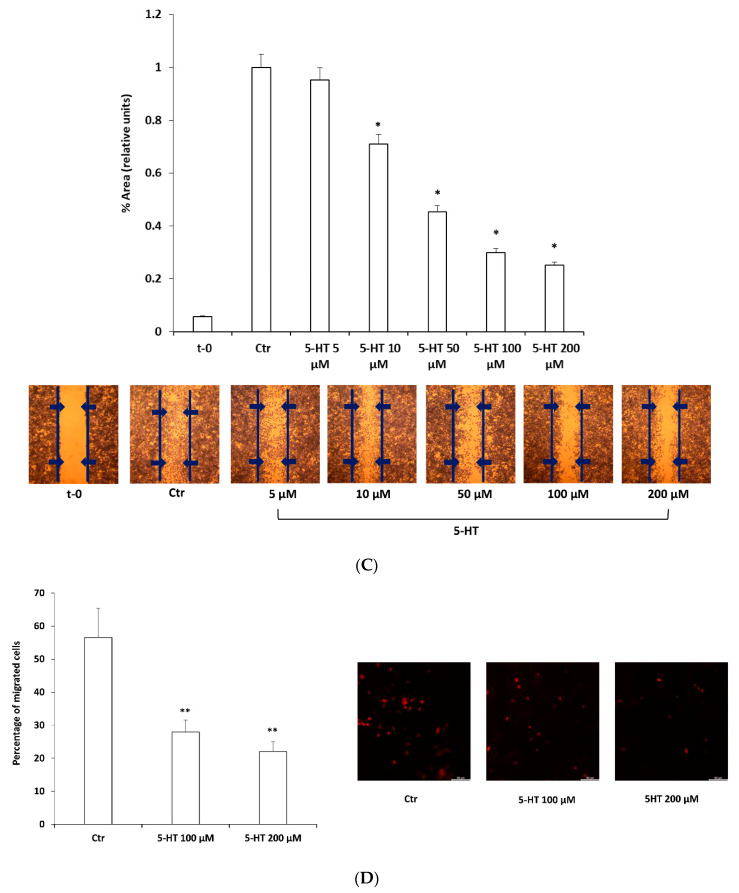
5-hydroxytryptamine (5-HT) inhibits M03-13 cell migration. (**A**) Semi-quantitative PCR analysis of 5-HT2A receptor in M03-13 and HEK-293 cells. The total extracted RNA was reverse transcribed and subsequently amplified by PCR 5-HT2AR specific primers. PCR analysis was performed with 39 cycles. M is the molecular weight indicator. (**B**) Immunoreactivity for 5-HT2AR in M03-13 cells was detected by cytofluorimetric analysis using primary rabbit antibodies against human 5-HT2AR and secondary rabbit IgG antibodies. The control was treated only with secondary antibodies. The insert shows the Western Blot for 5-HT2AR in M03-13 cells. (**C**) The migration of oligodendrocytes was studied by scratch analysis. The M03-13 cells were grown in 24-well plates in complete medium for 24 h; the following day, using a 100 µL pipette tip, a cut was made in the cell monolayer; immediately afterwards the cells were treated with different concentrations of 5-HT and allowed to migrate for 24 h. t-0 represents the sample to which the cut was made just before the analysis. The histogram shows the percentage values (mean ± SE) of the scratch area covered after 24 h from the application of the cut of three independent experiments performed in triplicate. In the lower part of panel C, a representative experiment is shown. (**D**) Effects of 5-HT on M03-13 cell migration by Fluoroblok^TM^ assay; on the right the images acquired by fluorescence microscopy; the histogram shows means ± SE values of three independent experiments relative to the control * *p* < 0.05; ** *p* < 0.01 vs. Ctr. The statistical analysis was performed with an ANOVA test.

It has been shown that rat primary OPCs express 5-HT1A and 5-HT2A subtypes of serotonin receptors [40]. Moreover, of particular interest in demyelinating diseases seems to be the 5-HT2AR which act as the glial cell receptor for the human polyomavirus JCV, the causal agent of progressive multifocal leukoencephalopathy, a fatal demyelinating disease developed in immunocompromised subjects [42]. For these reasons we first ascertain whether M03-13 cells, like primary rat OPCs, express 5-HT1A and 5-HT2A receptors subtypes by means of RT-PCR analysis. We found that M03-13 cells constitutively express 5-HT2A (Figure 1A) but not 5-HT1A receptor mRNA (not shown). Then, the expression of 5-HT2A receptor protein was evidenced by the immunofluorescence flow cytometry technique and by Western blot analysis (Figure 1B).

Next, we evaluated the effects of 5-HT on OPC migration, an early step of the whole OPC maturation process. Cell migration was assessed by scratch wound and FluoroBlok assay. The graph in Figure 1C, shows that 5-HT inhibits cell migration in a dose-dependent manner reaching the maximum effect at concentrations of 50–200 µM. The inhibitory effects of 5-HT on OPC migration were confirmed by FluoroBlok migration assay (Figure 1D). The effects of 5-HT on cell migration cannot be explained on the basis of the toxicity of the substance because it did not affect M03-13 cell survival evaluated by trypan blu assay. Indeed, cell viability even at 5-HT concentrations of 200 μM (89 ± 9.2%) was not significantly reduced compared to that of the control cells (97 ± 1.1%).

### 2.2. Protein Kinase C Mediates 5-HT Effects on M03-13 Cells Migration

**Figure 2:**
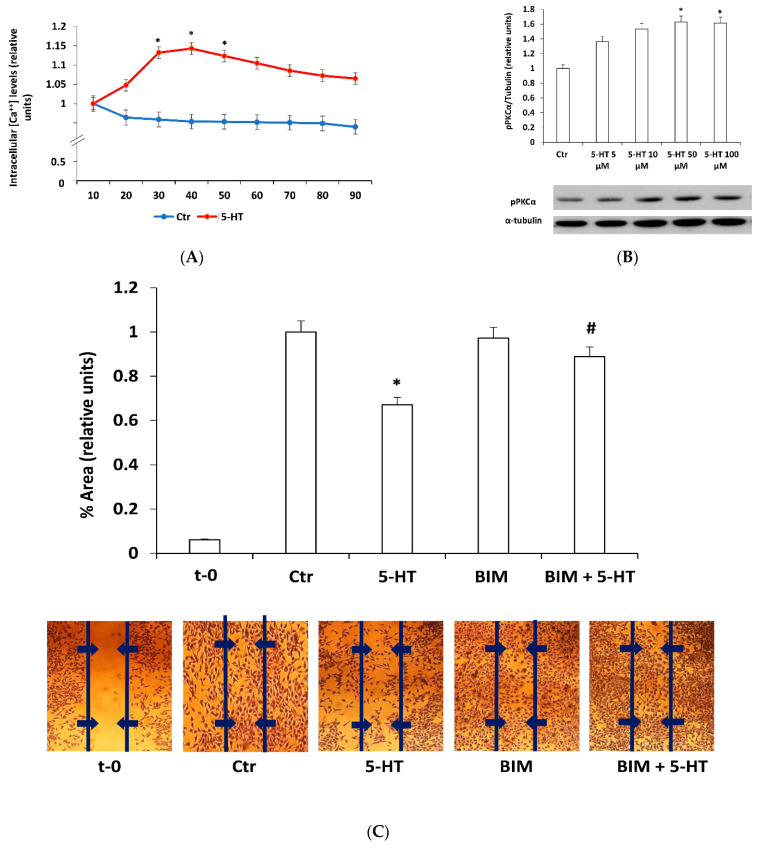
Protein kinase C (PKC) mediates 5-HT effects on M03-13 cell migration. (**A**) Time course of intracellular calcium levels in M03-13 cells stimulated with 5-HT 50 µM. The graph shows the mean values ± SE (n = 6) relative to control. (**B**) Western blotting analysis of P-PKCα levels in M03-13 cells incubated for 18 h in medium containing 0.2% Fetal Bovine Serum (FBS) and then stimulated with 5-HT for 30 min. The histogram shows the values (mean ± SE) obtained from the densitometric analysis of the protein bands normalized for α-tubulin in three independent experiments. A representative experiment is shown below the histogram. (**C**) Scratch analysis of M03-13 cells performed as indicated in Figure 1C. Immediately after the cut the cells were preincubated in the presence or absence of the PKC inhibitor, bis-indolyl-maleimide (BIM) (100 μM) for 30 min and then stimulated with 5-HT (50 μM) and allowed to migrate for 24 h. t-0 represents the sample that was cut immediately prior to analysis. The histogram shows the percentage values of the scratch area covered after 24 h after the application of the cut. On the lower part a representative experiment is shown. * *p* < 0.005 vs. Ctr, # *p* < 0.005 vs. 5-HT. The statistical analysis was performed with an ANOVA test (panels (**A**,**B**) and a *t*-test (panel **C**).

The 5-HT2A receptor activates PLC through Gαq signal transduction leading to increase in cytoplasmic IP3 and diacylglycerol (DAG), and consequent release of calcium from intracellular stores and protein kinase C (PKC) activation [43]. This cascade, the most important signaling pathway activated by 5-HT2AR, is active also in M03-13 cells. Indeed, we demonstrated that stimulation of M03-13 cells with 30–50 µM of 5-HT increases intracellular calcium concentration (Figure 2A) and phosphorylation levels of PKCα (Figure 2B). Treatment of cells with a bis-indolyl-maleimide (BIM), a PKC inhibitor, prevents the effects of 5-HT on cell migration, demonstrating PKC involvement in the mechanisms mediating the inhibitory effect of 5-HT on oligodendrocyte migration (Figure 2C).

### 2.3. ROS Mediate 5-HT Effects on M03-13 Cell Migration

**Figure 3:**
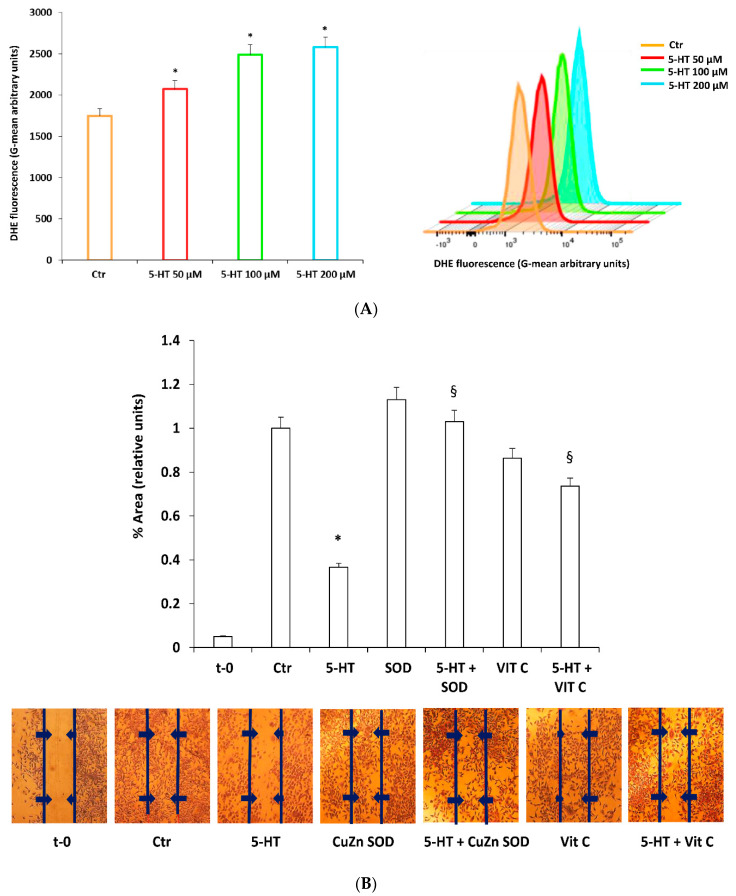
Reactive oxygen species (ROS) mediate 5-HT effects on M03-13 cell migration. (**A**) Cytofluorimetric determination of ROS levels. M03-13 were treated with 5-HT for 30 min, washed in phosphate-buffered saline (PBS) and resuspended in a serum-free medium containing 10 µM of dihydroethidium (DHE); 50,000 cells were analyzed by cytofluorimetry. (**B**) Scratch analysis of M03-13 cells was performed as indicated in Figure 1C. Immediately after the cut the cells were preincubated in the presence or absence of CuZnSOD (400 ng/mL) or vitamin C (1 μM) for 30 min and then stimulated with 5-HT (50 μM) and allowed to migrate for 24 h. t-0 represents the sample to which the cut was made just before the analysis. The histogram shows the percentage values of the scratch area covered after 24 h after application of the cut. A representative experiment is shown on the right. * *p* < 0.001 vs. Ctr, § *p* < 0.005 vs. 5-HT. The statistical analysis was performed with an ANOVA test (panel (**A**)) and a *t*-test (panel (**B**)).

Depending on the levels and on the site of production, ROS can be deleterious for cells or they can modulate many physiological processes including OPC differentiation [18]. Therefore, we evaluated the involvement of ROS in the inhibitory effects of 5-HT on OPC migration. Incubation of M03-13 with 50–100–200 µM of 5-HT produces an increase in reactive oxygen species (ROS) measured as Dihydroethidium (DHE) fluorescence by cytofluorimetry (Figure 3A).

To demonstrate the direct link between ROS production and cell migration, we evaluated the effect of antioxidant treatment of the cells. ROS increase plays an important role in blocking migration; indeed, the effects of 5-HT on the M03-13 cell migration are reverted by a generic antioxidant such as Vitamin C (Vit C). Moreover, we also evaluated the effects of enzymatic antioxidant Cu, Zn superoxide dismutase 1 (SOD1) [44,45,46]. The preincubation of cells with SOD1, like vitamin C, prevented 5-HT effects on cell migration (Figure 3B).

### 2.4. ROS Generated by NOXs Mediate 5-HT Induced Migration Block

**Figure 4:**
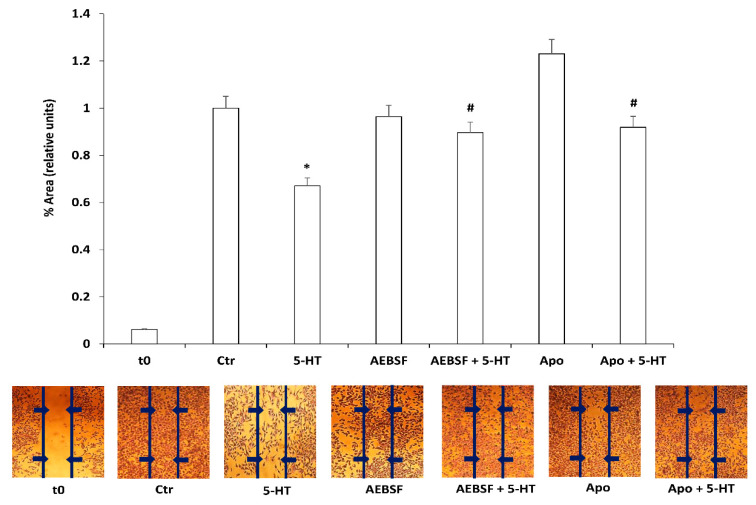
Scratch analysis of M03-13 cells performed as indicated in Figure 1C. immediately after the cut the cells were preincubated in the presence or absence of NOX inhibitors, AEBSF (40 µM) or apocynin (50 μM), for 30 min and then stimulated with 5-HT (50 μM) and allowed to migrate for 24 h. t-0 represents the sample that was cut immediately prior to analysis. The histogram shows the percentage values of the scratch area covered after 24 h after application of the cut. In the lower part a representative experiment is shown. * *p* < 0.001 vs. Ctr, # *p* < 0.005 vs. 5-HT. The statistical analysis was performed with a *t*-test.

NOX enzymes produce ROS in response to different signals. Among them 5-HT activates downstream signaling through ROS produced by NOX enzymes [47,48,49]. The NOX family consists of seven distinct members NOX1-5 and DUOX1-2, which share the ability to catalyze the production of a superoxide free radical by transferring one electron to oxygen from NADPH [23]. Among the members of the NOX enzymes family, M03-13 express the NOX3 and NOX5 isoforms [18]. To demonstrate the involvement of NOX-dependent ROS in 5-HT effects on oligodendrocytes migration, we used two NOX inhibitors, apocynin and 4-(2-aminoethyl)benzenesulfonyl fluoride (AEBSF). Scratch analysis showed that NOX inhibitors revert the effects of 5-HT on M03-13 migration, suggesting an involvement of NOX-generated ROS in the signaling pathways downstream of 5-HT2AR that leads to the blocking of oligodendrocyte migration (Figure 4).

### 2.5. 5-HT Inhibits M03-13 Cells Proliferation

**Figure 5:**
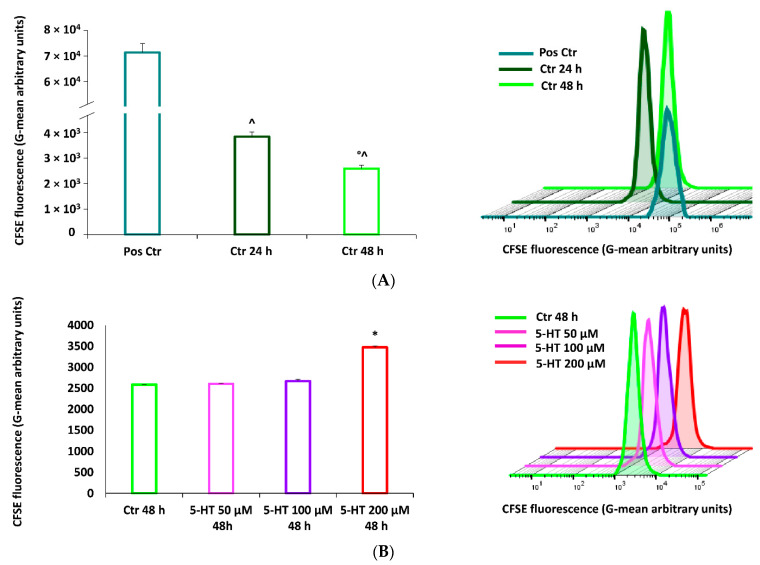

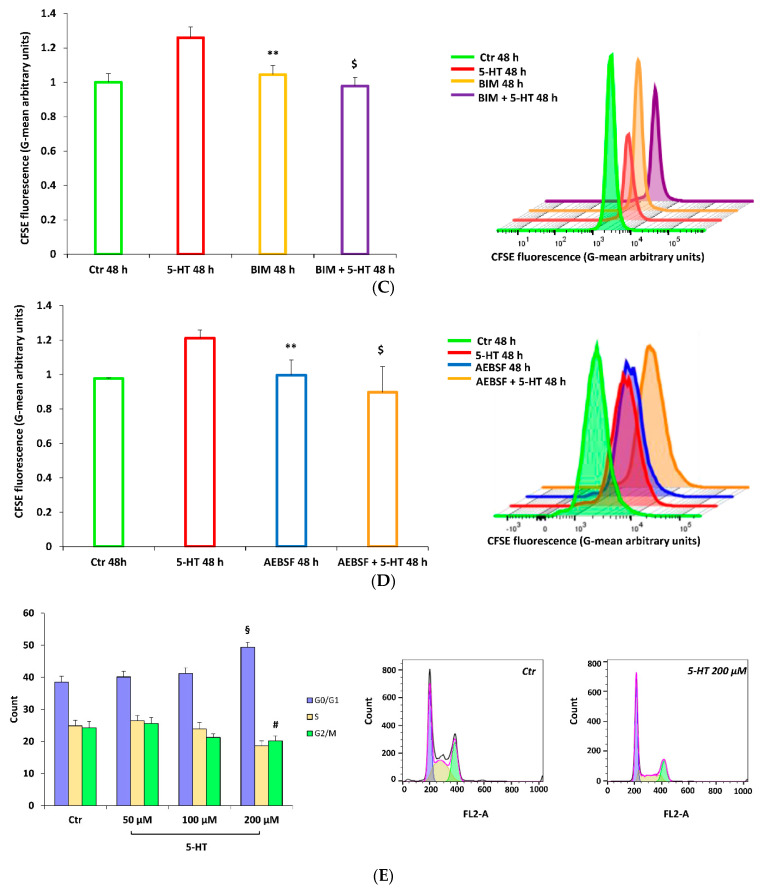
5-HT induces a proliferative block in M03-13 cells. (**A**) M03-13 were incubated with the succinimide carboxyfluorescein ester (CFSE) for 20 min, washed in PBS and platelets in complete medium. A total of 50,000 cells per point were analyzed by cytofluorimetry. The cells were analyzed 24 and 48 h after incubation with CFSE. The positive control indicates samples incubated with CFSE immediately before harvesting the cells for the analysis. (**B**) The cells were treated with and without 5-HT 50, 100 and 200 μM for 48 h in the presence of CFSE. (**C**,**D**) The cells were preincubated for 30 min with, BIM (10 µM) or AEBSF (10 µM) and then incubated with 200 uM of 5-HT for 48 h in the presence of CFSE. (**E**) Cell cycle analysis. M03-13 cells were treated with 5-HT for 24 h; the next day they were washed with PBS and treated with propidium iodide (PI) for 30 min; 10,000 cells were analyzed with the cytofluorimeter. The histograms show the averages of three experiments. The figure shows the means ± SE of three independent experiments; on the right the graphs of representative experiments are shown. ^ *p* < 0.01 vs. Pos Ctr, ° *p* < 0.01 vs. Ctr 24 h, * *p* < 0.05 vs. Ctr 48 h, ** *p* < 0.005 vs. Ctr 48 h, $ *p* < 0.05 vs. 5-HT 48 h, § *p* < 0.005 vs. Ctr G0/G1, # *p* < 0.005 vs. Ctr G2/M. The statistical analysis was performed with an ANOVA test (panels **A**, **B** and **D**) and with a *t*-test (panel **C**).

A further step in the maturation process of OPC is cell proliferation. The effects of 5-HT on M03-13 proliferation were detected by flow cytometric carboxyfluorescein ester (CFSE) fluorescent dye tracking. CFSE is a lipophilic fluorescent molecule capable of spreading freely through the cell membrane. Within the cell, acetate groups are removed from esterases, converting the CFSE into a molecule no longer able to cross the membrane. During cell division, therefore, each daughter cell will inherit exactly half the amount of CFSE that is present in the mother cell, allowing the number of cell divisions to be monitored by cytofluorimetric analysis (Figure 5A). Figure 5B shows that 5-HT inhibits by 34% proliferation after 48 h incubation at a dose of 200 µM. 5-HT blocks proliferation through the modulation of PKCα and NOX; indeed, in the presence of specific inhibitors, BIM and AEBSF, a reversion of the inhibitory effects of 5-HT on cell proliferation is observed (Figure 5C). We further studied the effects of 5-HT on the cell cycle observing a blockage in the G0/G1 phase and a reduction in the S phase of DNA synthesis in the presence of 5-HT (Figure 5D). These data confirm the inhibitory effect of 5-HT on oligodendrocytes proliferation.

### 2.6. 5-HT Mimics the Effects of PMA on Oligodendrocyte Phenotypic Markers in M03-13 Cells

**Figure 6:**
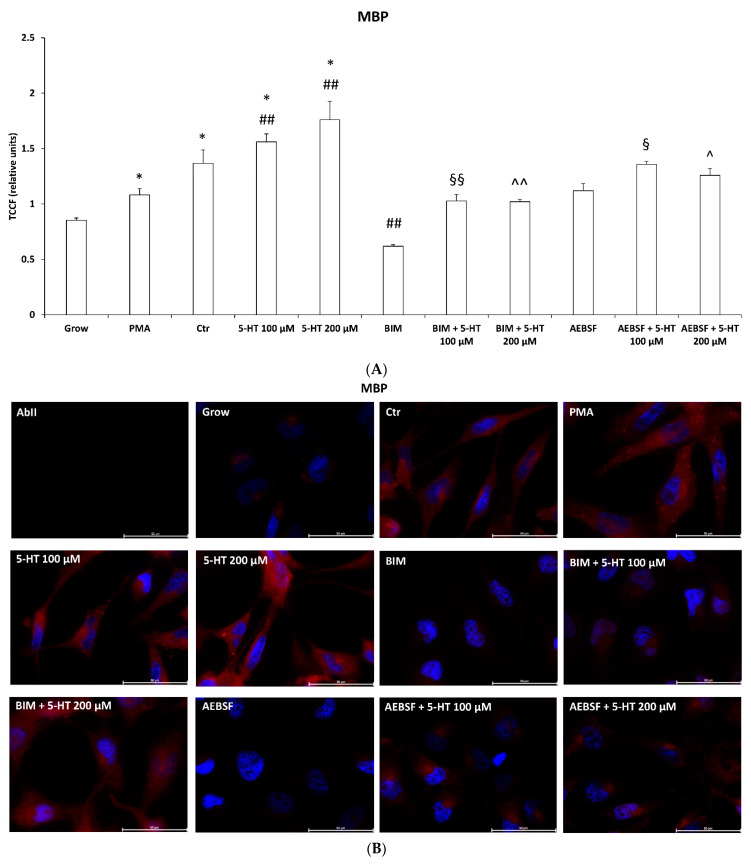

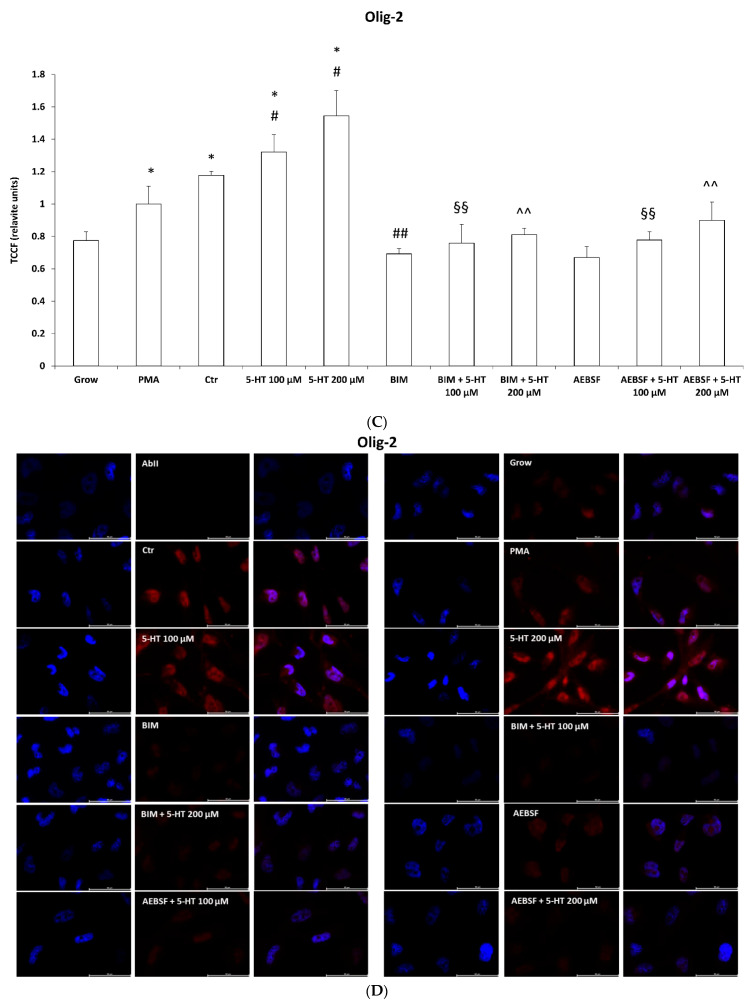
5-HT mimics oligodendrocyte differentiation stimulus inducing immunofluorescence microscopy levels of myelin basic protein (MBP) (**A**,**B**) and Olig-2 (**C**,**D**) in M03-13 cells. The cells were grown in medium without serum in the absence (Ctr) or presence of 100 nM phorbol 12-myristate 13-acetate (PMA)) or 100 and 200 μM 5-HT for 24 h. The inhibitors BIM (100 μM) and AEBSF (100 μM), were added in medium without serum 30 min prior to 5-HT stimulation. Cells were stained with nuclear dye 4,6-diamidino-2-phenylindole (DAPI) and with primary anti human MBP antibodies or anti human Olig-2 antibodies and CY3-conjugated anti rabbit IgG as secondary antibodies. Ab II was treated with secondary antibodies and DAPI alone. Grow indicates cells cultured in complete medium. In the immunoreactivity images of Olig-2 (**D**) three panels are shown: on the left nuclei (**blue**); on the center Olig-2 (**red**); on the right the merged image. The histograms (**A**, **C**) show the mean ± SE total corrected cellular fluorescence (TCCF) values obtained by quantitative analysis of 50 cells for each sample from three independent experiments performed in triplicate. * *p* ≤ 0.01 vs. grow; # *p* < 0.05. ## *p* ≤ 0.01 vs. Ctr; § *p* < 0.05, §§ *p* < 0.01 vs. 5-HT 100 µM; ^ *p* < 0.05, ^^ *p* < 0.01 vs. 5-HT 200 µM. The statistical analysis was performed with an ANOVA test. The images for quantitative analysis were acquired with a 20× objective. Panels B and D show representative images acquired with a 63× objective.

Oligodendrocyte differentiation and myelination are characterized both in vivo and in vitro, by sequential and synchronized expression of specific markers. To evaluate the effect of 5-HT on the late maturation phases of M03-13, we assessed the effects of the substance on two oligodendrocyte phenotypic markers, myelin basic protein (MBP) and Olig-2 by immunofluorescence microscopy. MBP is the major myelin protein necessary for the multilamellar myelin sheath formation and is a marker of terminal oligodendrocyte differentiation [50], while Olig-2 is the basic helix–loop–helix transcription factor, an early marker of oligodendrocyte specification regulating oligodendroglia formation [51,52]. Moreover, in mouse, Olig-2 overexpression enhanced OPCs differentiation and remyelination [53]. Olig-2 is expressed during all the stages of OPC maturation [54]; however, Olig-2, as well as MBP, is induced in M03-13 cells grown in the absence of serum or treated with 100 nM PMA in the absence of serum, which represent well established differentiation stimuli for these cells [18,55]. In cells grown in the absence of serum, the addition of 5-HT further enhanced MBP and Olig-2 fluorescence and cell morpholgy changed with an increase in process extentions. The effects of 5-HT on MBP and Olig-2 levels and cell morphology were inhibited in the presence of the PKC inhibitor BIM or NOX-inhibitor AEBSF, indicating that, even in this case, the effects of 5-HT are mediated by the PLC/PKC/NOXs pathway (Figure 6).

### 2.7. Mitochondrial Function and Oxidative Stress

**Figure 7:**
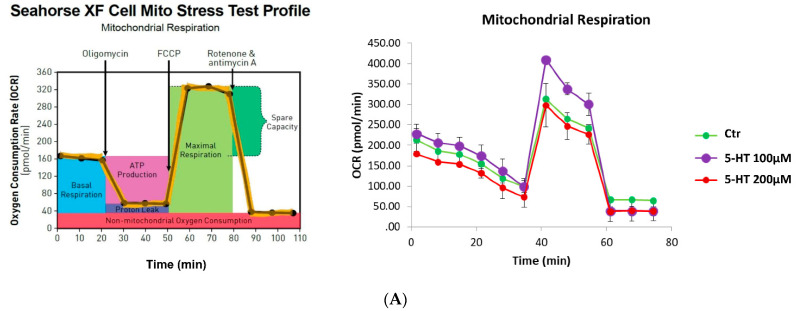

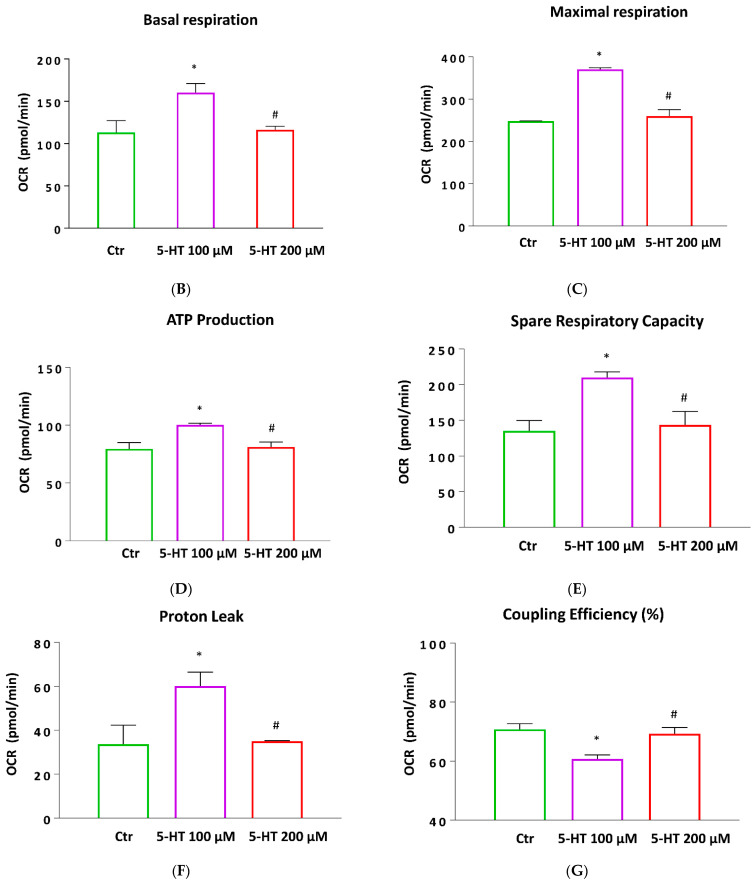
Effects of 5-HT on oxygen consumption rate (OCR) performed by Seahorse XFp analyzer in M03-13 cells. (**A**) Representative seahorse time courses of OCR; where indicated in the left panel, the following compounds were added: oligomycin, carbonyl cyanide 4-(trifluoromethoxy)phenylhydrazone (FCCP), rotenone plus antimycin A. Each point in the OCR time courses is the average SE of four technical replicates. Basal respiration (**B**), maximal respiration (**C**), ATP production (**D**), spare respiratory capacity (**E**), proton leak (**F**) and coupling efficiency (**G**) are reported. Data are presented as means ± ES. * *p* < 0.05 vs. Ctr, # *p* < 0.05 vs. 5-HT 100 μM. The statistical analysis was performed with an ANOVA test.

Since oligodendrocytes also provide metabolic support to neurons, we evaluated the effects of 5-HT on mitochondrial activity. M03-13 oligodendrocytes were stimulated with 100–200 µM 5-HT and the mitochondrial function was examined using the Seahorse XFp cell mito stress kit (Seahorse Bioscience, North Billerica, MA, USA). It was performed in real-time at basal level and following a sequential addition of mitochondrial respiration inhibitors: oligomycin, carbonyl cyanide 4-(trifluoromethoxy)phenylhydrazone (FCCP), and a combination of antimycin A and rotenone Oligomycin, an inhibitor of ATP synthase, was used to distinguish between oxygen consumption that cells use to synthesize ATP (ATP-linked respiration) and oxygen consumption that is used to overcome the proton leak across the mitochondrial membrane. FCCP treatment collapses the proton gradient and disrupts the mitochondrial membrane potential, which allows measurement of the maximal uncoupled respiration (maximal respiration). A combination treatment of rotenone, a complex I inhibitor, and antimycin A, a complex III inhibitor, was used to shut down mitochondrial respiration, which enables differentiation between the mitochondrial (basal respiration) and non-mitochondrial respiration contribution to total respiration. The difference between maximal and basal respiration constitutes the spare capacity (Figure 7A).

The results showed, in M03-13 oligodendrocytes stimulated with 5-HT 100 µM, an increase not only in the basal respiration (Figure 7B) but also in the maximal respiration measured after injection of the uncoupler agent FCCP, compared to control and M03-13 oligodendrocytes stimulated with 5-HT 200 µM (Figure 7C). These results are consistent with the increase in ATP production in the 5-HT 100 µM-treated cells compared to other cell groups (Figure 7D). The capacity of the cell to respond to increased energy demand or under stress, indicated as spare respiratory capacity, increased the 5-HT 100 µM-treated cells compared to other cell groups Moreover, a significant increase in proton leak and, as a consequence, a significant reduction in the coupling efficiency was observed in 5-HT 100 µM-treated cells compared to control or 5-HT 200 µM-treated cells (Figure 7E–G).

In addition, mitochondrial oxygen consumption rates were also polarographically evaluated, by using a Hansatech respirometer, in mitochondria isolated from different experimental groups.

**Figure 8:**
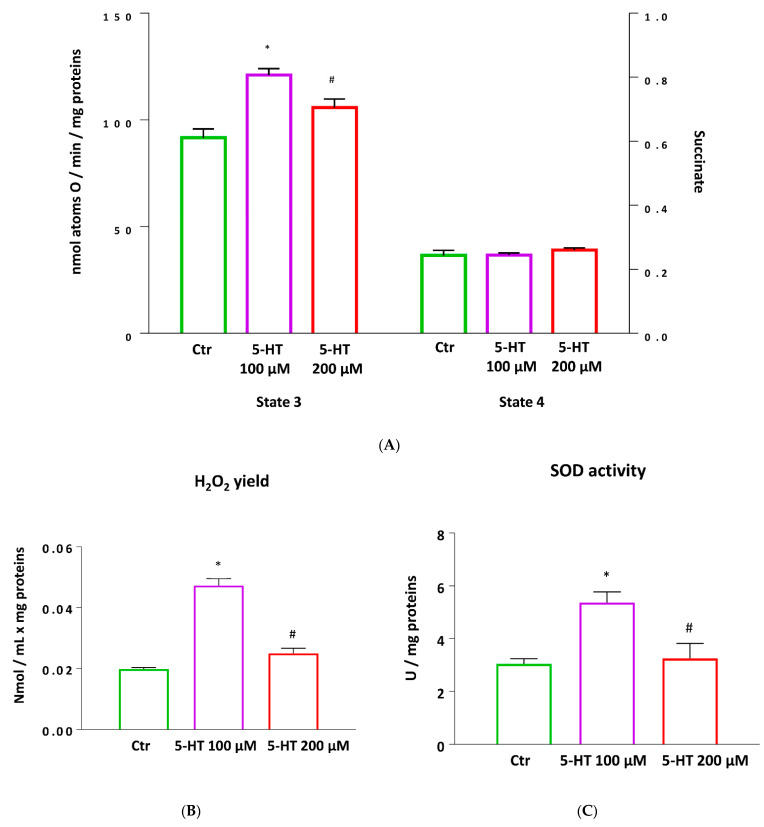
Effect of 5-HT 100 μM and 200 μM on isolated mitochondria from M03-13 cells monitored by Hansatech respirometer. Mitochondrial respiration rates measured in the presence of succinate, rotenone and Adenosine diphosphate (ADP) (state 3) and in the absence of ADP (state 4) (**A**), H_2_O_2_ yield (**B**) and SOD activity (**C**) are reported. Data are presented as means ± ES. * *p* < 0.05 vs. CTR, # *p* < 0.05 vs. 5-HT 100 μM.

The highest state 3 respiration rate observed in mitochondria of the oligodendrocytes stimulated with 5-HT 100 µM, confirms the effect of the stimulation with 5-TH 100 µM on the increase in the ATP production when ADP was supplied in presence of succinate as substrate. No variation in state 4 was detected between groups (Figure 8A).

In order to explore whether the changes in mitochondrial function resulted in alterations of mitochondrial ROS production, H_2_O_2_ release and SOD activity were measured in mitochondria isolated from M03-13 cells. A significantly higher both H_2_O_2_ production and SOD activity was observed in oligodendrocytes stimulated with 5-TH-100 µM than in control or 5-TH-200 µM stimulated cells (Figure 8B,C).

## 3. Discussion

Our data indicate that the activation of the 5-HT receptor/PKC/NOX cascade generates ROS that inhibit M03-13 migration. Moreover, 5-HT inhibits M03-13 proliferation inducing cell cycle arrest in the G0/G1 phase committing cells toward differentiation via PKC/NOX-dependent ROS production. 5-HT also modulates mitochondrial respiration and antioxidant activity. Altogether, these data highlight an important role of 5-HT signaling in the maturation process and mitochondrial function of M03-13. These data suggest that similar process might occur during OPC maturation, although it has been shown that the M03-13 cell line recapitulates only some features of OPC maturation [56].

Oligodendrocytes express multiple neurotransmitter receptors and transporters [57,58]. Different neurotransmitters modulate the maturation process of oligodendrocytes adapting axonal activity to myelination. Adenosine modulates late phases of oligodendrocyte development stimulating myelin formation [59]. Glutamate has been shown to inhibit OPC proliferation through α-amino-3-hydroxy-5-methyl-4-isoxazolepropionic acid (AMPA) and kainate receptors activation [60,61]. The inhibitory effect of glutamate on OL proliferation has been explained [62] as an event needed to decrease the number of oligodendrocytes once the precursors have reached their destination around the axons. Similarly, the inhibitory effects of serotonin on M03-13 migration and proliferation (Figure 1C and Figure 5B,D) suggest that 5-HT signaling plays a role in the advanced steps of the OPC maturation process when OPC exits the cell cycle and initiates a transcriptional program resulting in the differentiation into mature OLs. This hypothesis is sustained by our data demonstrating that 5-HT mimics the effects of differentiation stimuli (Figure 6) and by the finding that olanzapine, an antagonist of dopamine and serotonin receptors used as an antipsychotic drug, increases proliferation and inhibit differentiation of rat primary culture of oligodendrocytes [63].

5-HT, besides blocking M03-13 migration and proliferation, promotes the increase in oligodendrocyte phenotypic markers MBP and Olig-2 via the PKC pathway, mimicking the effects of differentiation stimuli like PMA (Figure 2C, Figure 5C and Figure 6) [18,51,53,55]. The increase in NOX-dependent ROS levels in 5-HT treated cells (Figure 3A) contribute to promote oligodendrocyte maturation since NOXs inhibitor AEBSF prevents 5-HT dependent increase in MBP and Olig-2 (Figure 6). Our data as well as that of other groups show that ROS induce OPC differentiation. Hydrogen peroxide induces oligodendrocyte differentiation in NSCs obtained from the subventricular zone [64] and ROS levels are correlated with the extent of differentiation [65]. Hydrogen peroxide mimics the effects of differentiation stimuli also in M03-13 cells via the PLC/PKC/NOX pathway [18].

Mitochondria activity is necessary for differentiation, a highly energy-consuming event. Actually, mitochondrial dysfunction has been often associated to demyelination [66,67] and the impairment of oligodendrocyte differentiation induced by TNFα has been causally linked to mitochondria deficiency in primary rat OPC [68]. We showed that serotonin increases oxidative capacity in mitochondria isolated from 5-HT treated M03-13 cells (Figure 8). The higher state 3 respiration rate is closely related to a concomitant increase in H_2_O_2_ yield and SOD activity, suggesting that the balance of intracellular ROS levels is maintained by antioxidant enzyme activities [69,70]. To better characterize the metabolic state of 5-HT treated M03-13 cells, we then directly assessed the 5-HT effect on ATP production and energy efficiency using a Seahorse XF Analyzer. In agreement with data obtained in isolated mitochondria, Seahorse analyses evidenced in 5-HT-100-treated cells an increase in spare respiratory capacity, indicating the ability of the cell to respond to increased energy demand or when under stress (Figure 7). Moreover, this result is in line with an increase in mitochondrial uncoupling, as shown by higher proton leakage. The uncoupling is a major mechanism for the adjustment of the membrane potential to control mitochondrial ROS emission. By mildly uncoupling, the mitochondria can avoid the oversupply of electrons/reducing equivalents into the respiratory complexes and minimize the likelihood of electron interaction with oxygen [71]. All together these results suggest that ROS levels were balanced through the proton leakage mechanism and antioxidant enzyme activities.

5-HT increases ROS levels via NADPH oxidase in different biological systems. In colon epithelial cells 5-HT increases ROS levels by activating NOX2 and DUOX2 linking the changes in 5-HT content in the gut of patients with inflammatory bowel disease (IBD) to its pathogenesis [47]. NOX-dependent ROS are also implicated in the anorexigenic effects of 5-HT at the hypothalamic level [48]. Moreover, in neuroblastoma cells, 5-HT transactivates the platelet-derived growth factor (PDGF) type β receptor as well as the TrkB receptor through NADPH oxidase-dependent ROS production. Our data showing an increase in ROS levels (Figure 3A) in 5-HT-treated M03-13 confirm all this evidence. ROS have been proven both to inhibit [72,73] or promote cell migration [74,75]. Similarly, the effects of ROS in cells proliferation are conflicting [76]. We evidenced that 5-HT inhibition of M03-13 migration and proliferation as well as commitment towards differentiation, is mediated by ROS. In OPC, NOX-dependent ROS are thus mediators of the 5-HT effects. More specifically, our data, for the first time, show an involvement of NOX-derived ROS in the inhibitory effect of 5-HT on M03-13 migration and proliferation in favor of differentiation. In contrast, mtROS do not seem to mediate 5-HT effects on cell migration and proliferation, since the mitochondria-targeted antioxidant mitoTEMPO did not revert 5-HT effects (data not shown).

Although at low levels ROS may be the molecular mediators of physiological processes, high levels of ROS have been implicated in the pathogenesis of many diseases, including demyelinating diseases such as MS [77,78]. In the early phases of the disease, ROS production is crucial for the formation of local inflammatory lesions, contributing to the blood–brain barrier disruption, infiltration of leukocytes, microglia activation and oligodendrocyte cell death. In the progressive phase of the disease, ROS have been invoked as mediators of axonal damage and neurodegeneration [79,80]. Moreover, oxidative stress has also been involved in remyelination failure inside multiple sclerosis lesions [81]. Remyelination implies that, in response to damage, changes in microglia and astrocytes induce a switch of local OPC from a quiescent to a regenerative phenotype [82], thus assuring the repopulation of the demyelinating area. Increased levels of ROS prevent remyelination in MS lesions and substances with antioxidant properties have been proven to ameliorate remyelination [83,84].

Experimental evidence links the 5-HT pathway to multiple sclerosis. In Cerebrospinal fluid (CSF) of MS patients there are lower levels of the 5-HT metabolite, 5-hydroxyindoleacetic acid (5-HIAA) compared to control subjects, and their levels negatively related to the rate of disability accumulation in Relapsing-remitting MS (RRMS) [85]. Much evidence of an existing association between 5-HT homeostasis and demyelinating disease have been collected in animal models of multiple sclerosis as experimental autoimmune encephalomyelitis (EAE), characterized by variable degrees of demyelination induced by the immunization of susceptible animals with myelin antigens. The disease course in EAE knockout mice for the 5-HT transporter were attenuated compared to wildtype control mice [86]; moreover, increased 5-HT levels and its reduced turnover was observed in EAE obese mice [87] and treatment with risperidone, an antipsychotic drug, reduces the size and number of spinal cord lesions in EAE animals [88].

Our data showing a mechanistic link between serotonin and oligodendrocyte maturation processes support the hypothesis that a dysregulation of serotonin system leading to oxidative stress, could contribute to the onset of MS lesions and to the failure of remyelination giving rise to progressive demyelination and neurodegeneration which results in the accumulation of disability. Therefore, 5-HT receptors and signaling molecules downstream are promising therapeutic targets for the treatment of MS.

## 4. Materials and Methods

### 4.1. Cell Cultures

The M03-13 cells (CELLution Biosystem Inc., Paletta Crt, Burlington, Canada) were derived from the fusion of a 6-thioguanine-resistant mutant of a human rhabdomyosarcoma with OLs obtained from adult human brain, these cells are an immortal human–human hybrid cell line with the phenotypic characteristics of primary OLs. Upon serum starvation and/or PMA stimulation, these cells undergo increase in process extentions, decrease in proliferation and increased expression of genes associated with mature oligodendrocytes such as MBP [55,56,89]. The M03-13 cells were grown in Dulbecco’s Modified Eagles Medium (DMEM; GIBCO Invitrogen), containing 4.5 g/L glucose (GIBCO, Auckland, New Zealand), supplemented with 10% Fetal Bovine Serum (FBS; Sigma-Aldrich, St. Louis, MI, USA), 100 U/mL penicillin and 100 μg/mL streptomycin. The cells were kept in a 5% CO_2_ and 95% air atmosphere at 37 °C. M03-13 were always used within 15th passage.

### 4.2. Intracellular Calcium Flux Assay

Intracellular calcium levels in M03-13 cells were measured using the Fluo-4 NW calcium indicator kit (Life Technologies, Carlsbad, CA, USA), according to the manufacturer’s instructions, as previously described [90]. Briefly, M03-13 cells were grown in 96-well microplates (30,000/w) for 18 h, then were loaded with the Fluo-4 probe in the presence of 5 mM Probenecid for 30 min at 37 °C and then 30 min at room temperature. Fluorescence was measured using Fluoroskan Ascent (Thermo electronic corporation, Waltham, MA, USA), a fluorescent plate reader with excitation at 485 nm and emission at 538 nm. Fluorescence was recorded every 6 s recorded for 1 min before loading cells with 50 µM 5-HT and was then monitored for an additional 10 min. Blank samples of fluorescence were also measured and subtracted from all experimental points. Data were analyzed by Ascent software (Waltham, MA, USA).

### 4.3. Western Blotting Analysis

M03-13 cells, grown to semiconfluence in 100-mm dishes, were incubated for 18 h in 0.2% FBS medium. Lysates of these cells were obtained in RIPA buffer containing: 50 mM TrisHCl, pH 7.5, 150 mM NaCl, 1% NP40, 0.5% deoxycholate, 0.1% sodium dodecyl sulphate (SDS), 2.5 mM Napyrophosphate, 1 mM β-glycerophosphate, 1 mM NaVO_4_, 1 mM NaF, 0.5 mM phenyl-methyl-sulfonyl-fluoride (PMSF), and a cocktail of protease inhibitors (Roche Applied Bioscience Penzberg, Upper Bavaria, Germany ). The cells were kept for 15 min at 4 °C and distrupted by repeated aspiration through a 21-gage needle. Cell lysates were centrifuged for 10 min at 13,000 rpm and the pellets were discarded. Fifty micrograms of total proteins were subjected to SDS—10% polyacrylamide gel electrophoresis (SDS-PAGE). After, the proteins were transferred with a Trans-Blot Cell (Bio-Rad Laboratories, Berkeley, CA, USA) in transfer buffer (25 mM Tris, 192 mM glycine, 20% methanol) onto a nitrocellulose filter membrane (GEHealthcare, Amersham PI, UK). The membranes were placed in 5% non-fat milk in tris buffered saline, 0.1% Tween 20 (TBST, Bio-Rad Laboratories Segrate Milan Italy) at 4 °C for 18 h to block the non-specific binding sites. Filters were incubated with specific rabbit polyclonal antibodies against p-PKCα (Ser657) (Upstate) or 5-HT2AR (Abcam) and then incubated with a peroxidase conjugated anti-rabbit secondary antibody (GEHealthcare, Amersham, UK).

Peroxidase activity was detected with the enhanced chemiluminescence (ECL) system (GE-Healthcare). Then the membrane was stripped and reprobed with an anti α-tubulin antibody (Sigma-Aldrich, St. Louis, MI, USA) for the normalization. Protein bands were revealed by ECL and, when specified, quantified by densitometry using ImageJ software.

### 4.4. CFSE Assay

The oligodendrocytes (M03-13 cells) grown to semiconfluence in 100-mm Petri dishes at confluence were trypsinized and 1 × 10^6^ cells were resuspended in complete medium containing CFSE 5 µM; then, cells were incubated for 20 min in an incubator at 37 °C in the dark. Next, the cells were washed in PBS buffer to remove the excess of CFSE and resuspended in complete medium (DMEM) in the presence or in absence of 10, 50, 100 and 200 µM serotonin (5-HT). After 24 h or 48 h the M0313 cells were trypsinized, centrifuged for 5 min at 1000× *g*, washed and resuspended in PBS Buffer, then the samples were analyzed by flow cytometry using FACSCAN (BD, Heidelberg, Germany) and the data analyzed by FlowJo software.

### 4.5. Cell Cycle Analysis

For the cell cycle analysis, cells were starved in medium containing 0.2% serum for 18 h and then stimulated overnight with 50, 100 and 200 µM serotonin. The cell pellets were then incubated with 300 µL of PI Buffer (Triton 0.1%, RNase A 0.1 mg/mL, Propidium Iodide 10 µg/mL diluted in PBS buffer) for 30 min in agitation. Samples were read at FACSCAN (BD, Heidelberg, Germany) and data analysis was performed with the FlowJo software.

### 4.6. DHE (Dihydroethidium) Analysis

Reactive oxygen species levels were determined by flow cytometry using fluorescent probes, Dihydroethidium (DHE) (Molecular Probes Eugene, OR, USA)^.^ M03-13 cells were grown to semi-confluence in 60-mm culture dishes and incubated for 18 h in medium containing 0.2% FBS. An amount of 5 × 10^5^ M03-13 cells were grown in 60-mm Petri dishes and incubated for 18 h in medium containing 0.2% FBS. The day after cells were stimulated for 30 min with 50/100/200 µM of 5-HT. After incubations, the cells were trypsinized and washed twice with PBS and incubated with DHE (10 μM) for 30 min at 37 °C. The cells were analyzed using a FACSCAN flow cytometer (BD, Heidelberg, Germany) and FlowJo software.

### 4.7. Scratch Assay

The Scratch Assay or Wound Healing Assay is used for the evaluation of cell migration in vitro. A scratch is performed in this assay with a 100-μL Gilson pipette tip on a 70–80% semi-confluent monolayer of cells. The Scratch Assay was performed on 30,000 M03-13 cells grown in 24 multi-well plates. The next day, the cells were washed with PBS, and then these were incubated with 5-HT at increasing concentrations of 1, 5, 10, 50 and 100 µM in a complete medium. After 24 h, the medium was removed and cells immediately fixed in 3.7% Paraformaldehyde 15 min at room temperature (22 °C) and, after two washes in PBS, the cells were stained with Coomassie Blue for 30 min at room temperature and washed with distilled water. To visualize the result of the scratch assay, digital images of the wells were acquired by optical microscopy and quantitatively analyzed with Image J software. The quantitative analysis uses the signal intensity of the pixels to count the number of cells present in the cut area (% area) in relation to the cells present within the total image area.

### 4.8. Cell Migration Assay with FluoroBlok™

FluoroBlok™ cell culture inserts (Invitrogen Carlsbad, CA, USA) are fitted with polyethylene terephthalate (PET) membrane, which blocks the transmission of visible light between 490 and 700 nm. The cells are placed in the upper chamber, while the lower chamber contains the complete medium acting as an attractant. If able to migrate, the cells will cross the microporous membrane. The migrated cells, treated with a fluorescent probe, can be visualized on the opposite side of the membrane with a fluorescence microscope. Fluoroblok^TM^ inserts were placed on the 24-well multi-well plate and rehydrated with smooth DMEM for 2 h in a 37 °C incubator at 5% CO_2_. At total of 2.5 × 10^4^ cells per spot were suspended in serum-free DMEM in the presence and absence of 100 and 200 µM serotonin (5-HT) and placed in the upper chamber of the insert. The lower face of the membrane was immersed in DMEM enriched with 10% FBS. After incubation for 18–20 h, the culture medium was gently aspirated from the insert, and the cells were fixed with 3.7% paraformaldehyde for 20 min at room temperature and incubated for another 20 min at room temperature with the fluorescent nuclear probe DRAQ5^TM^ (BioLegend, San Diego, CA, USA).

The blue PET membrane was then removed from the inserts and fixed on a slide, and the upper (non-migrated cells) and lower (migrated cells) faces of the membrane were observed under a fluorescence microscope (Leica DMi8 Mycrosistems CMS GmBH, Milan, Italy). Microscopic images of both sides of the membrane of each sample were taken, and the number of cells was quantified using ImageJ. Analysis of the results was carried out by assessing the number of migrated cells compared to the total number.

### 4.9. RNA Extraction and RT-PCR

Total RNA was extracted using TRI-Reagent according to the protocol provided by the manufacturer (Sigma-Aldrich, St. Louis, MO, USA). Total RNA (1 µg) was reverse transcribed with the SensiFASTcDNA Synthesis Kit (Bioline—Meridiam Bioscience Aurogene, Rome, Italy) by oligo-dT primers for 35 min in 20 µL reaction volumes.

Semi-quantitative PCR was performed using the FastStart Taq DNA Polymerase kit (Roche Applied Bioscience Penzberg, Upper Bavaria, Germany) in 20 µL final volume containing 0.5 mM deoxynucleotide triphosphates (dNTP), 0.2 µM of the specific primers and 100 ng of sample cDNA. The PCR conditions used were 95 °C 5 min, (95 °C 50 s, 57 °C 45 s, 72 °C 1.50 min) and 72 °C 7 min. The reactions were carried out at cycle number 39. Primers used in these experiments are shown in Table 1. Samples were loaded on 2% agarose gels with ethidium bromide. The marker that was used for fragment measurements was a 100-bp DNA Ladder (Fisher Molecular Biology, Waltham, MA, USA).

### 4.10. Immunofluorescence Microscopy

M03-13 cells were grown on glass coverslips under culture conditions described in the specific experiments. For MBP staining, the cellular medium was removed and cells immediately fixed in 3.7% paraformaldehyde in PBS with 2% sucrose, pH 7.4, for 5 min at 22 °C, and, after two washes in PBS with 2% Sucrose, permeabilized for 10 min at 4 °C with 0.01% saponin (Sigma-Aldrich, from quillaja bark) in PBS. The cells, after blocking with 20% FBS in PBS with 0.01% saponin for 30 min at 4 °C, were labeled with primary rabbit polyclonal anti human MBP antibody (Millipore Upstate). The cells were washed and labeled with secondary Cy3-conjugated anti-rabbit IgG (Jackson ImmunoResearch, West Grove, PA, USA). Controls were incubated with secondary antibodies alone.

For Olig-2 staining, the medium was removed and cells immediately fixed in 3.7% paraformaldehyde and then permeabilized for 5 min at 4 °C with 0.1% Triton X-100 in 20 mM Hepes, 300 mM sucrose, 50 mM NaCl, 3 mM MgCl_2_. The cells, after blocking with 20% FBS in PBS for 30 min at 4 °C, were labelled with primary rabbit polyclonal anti human Olig-2 antibody (Santa Cruz). The cells were washed and labeled with secondary Cy3-conjugated anti-rabbit IgG (Jackson ImmunoResearch, USA). Controls were incubated with secondary antibodies alone. For both stainings, the cells were treated with 4,6-diamidino-2-phenylindole (DAPI), a nuclear marker, and then the coverslips were briefly washed, first in PBS and then in distilled water, and finally mounted on glass slides for microscopy examination. Cells were analyzed with a Leica DMi8 microscope. Afterwards, images were analyzed using the ImageJ software according to the protocol used by McCloy et al. [91]. Briefly, a line was drawn around each individual cell to calculate the area and intensity of emitted fluorescence (integrated density). The reported values were normalized to the field background. The total corrected cellular fluorescence (TCCF) was calculated using the following formula:

(TCCF) = integrated density − (area of selected cell × mean fluorescence of background readings)(1)

The mean value of TCCF was obtained from the analysis of 50 cells for each sample from three independent experiments performed in triplicate.

### 4.11. Seahorse XFp Analyses

Cellular oxygen consumption measurements in M03-13 cells were performed by the Seahorse XFp analyzer (Seahorse Biosciences, North Billerica, MA, USA), by using the Cell Mito Stress Test kit (cat# 103010-100). Control cells and 5-HT treated cells (100 and 200 µM) were seeded (30.000 cells/well) in Seahorse mini plates in complete medium for 12 and then it was removed, and the cells were replaced with medium containing 0.2% FBS. Before cell mito stress analyses, the medium was replaced with a buffered base medium (Agilent Seahorse-103193) supplemented with 2 mM glutamine, 1 mM pyruvate and 10 mM glucose at pH 7.4 and equilibrated at 37 °C in a CO_2_ free incubator for at least 1 h. Basal oxygen consumption rate (OCR) was determined in the presence of glutamine (2 mM) and pyruvate (1 mM). The proton leak was determined after inhibition of mitochondrial ATP production by 1 µM oligomycin, as an inhibitor of the F0-F1 ATPase. Furthermore, the measurement of the ATP production in the basal state was obtained from the decrease in respiration by inhibition of the ATP synthase with oligomycin. Afterward, the mitochondrial electron transport chain was stimulated maximally by the addition of the uncoupler FCCP (1 µM). Finally, the extra-mitochondrial respiration was estimated after the addition of the inhibitors of the complexes I and III, rotenone A (0.5 µM) and antimycin (0.5 µM). Coupling efficiency is the proportion of the oxygen consumed to drive ATP synthesis compared with that driving proton leakage and was calculated as the fraction of basal mitochondrial OCR used for ATP synthesis (ATP-linked OCR/basal OCR). Spare capacity is the capacity of the cell to respond to an energetic demand and was calculated as the difference between the maximal respiration and basal respiration. The mitochondrial respiration was expressed as the oxygen consumption rate per minute normalized to the number of cells. The data normalization of OCR can be performed in different ways to minimize inconsistency and variations from well-to-well [92]. In our experimental conditions, the same cell number/well was plated before the OCR measurements; the cell count was obtained by using the Burker chamber. Cell counting is a non-destructive method to generate data for Seahorse normalization. In addition, at the end of analyses, we determined the protein content/well by Bradford assay without observing significant differences.

### 4.12. Mitochondria Isolation

Mitochondria isolation was carried out using minor modifications to procedures described by Frezza et al. [93]. Briefly, the medium was removed, and the cells were washed twice with PBS by centrifugation at 1200 rpm at 4 °C for 10 min. After removing the supernatant, the pellet was resuspended in 1 mL of the buffer for isolation (IB-cells solution) containing 225 mM mannitol, 75 mM sucrose, 0.1 mM ethylene glycol-bis(β-aminoethyl ether)-N,N,N′,N′-tetraacetic acid (EGTA), 30 mM Tris HCl and was homogenized with the above medium in a Potter Elvehjem homogenizer (Heidolph, Kelheim, Germany) set at 300 rpm (3 strokes, 30 s). Afterward, the homogenate was centrifuged at 300× *g* for 10 min at 4 °C. The supernatant was collected and centrifuged at 7000× *g* for 12 min, the pellet was washed once and resuspended in IB cells medium and centrifugated at 7000× *g* for 12 min. The resulting pellet, containing mitochondria, was washed and resuspended in IB cells. The protein content of the mitochondrial suspension was determined by the method of Hartree [94] using bovine serum albumin (BSA) as the protein standard. Isolated mitochondria were then used for the determination of respiratory parameters.

Mitochondrial oxygen consumption was polarographically monitored by a Hansatech respirometer at 30 °C. Isolated mitochondria (0.1 mg protein/mL) were incubated in 1 mL of incubation medium containing 80 mM KCl, 50 Mm HEPES, 1 mM Ethylenediaminetetraacetic acid (EDTA), 5 mM KH_2_PO_4_, pH 7.0, and 0.1% (w/v) fatty acid-free BSA. In the presence of 10 mM succinate, 3.75 µM rotenone and 0.6 mM ADP, state 3 oxygen consumption was measured. State 4 was obtained in the absence of ADP. The respiratory control ratio (RCR) was calculated as the ratio between states 3 and 4.

### 4.13. H_2_O_2_ Release

Rate of mitochondrial H_2_O_2_ release was assayed by following the linear increase in fluorescence (excitation at 312 nm and emission at 420 nm) due to the oxidation of homovanillic acid in the presence of horseradish peroxidase [95].

### 4.14. SOD Activity

Superoxide dismutase (SOD) specific activity was measured in a medium containing 0.1 mM EDTA, 2 mM KCN, 50 mM KH_2_PO_4_, pH 7.8, 20 mM cytochrome c, 5 mM xanthine, and 0.01 U of xanthine oxidase. Determinations were carried out spectrophotometrically (550 nm) at 25 °C, by monitoring the decrease in the reduction rate of cytochrome c by superoxide radicals generated by the xanthine–xanthine oxidase system. One unit of SOD activity is defined as the concentration of enzyme that inhibits cytochrome c reduction by 50% in the presence of xanthine and xanthine oxidase [96].

### 4.15. Cell Viability Assay

The toxicity of 5-HT treatment on M03-13 cells was tested by Trypan Blue staining. A total of 2.3 × 10^5^ cell plated in 35-mm Petri dishes, were treated with increasing doses of 5-HT (50, 100 and 200 μM) for 18 h in medium without serum. After trypsinization and washing in PBS, the cells were suspended in diluted Trypan Blue (1:1 with PBS) and then immediately counted in Burker’s chamber. The viable (unstained) and unviable (stained) cells were counted separately. The percentage of viable cells was calculated as follows: Cell Viability (%) = 1 − (number of nonviable cells/total number of cells) × 100.

### 4.16. Statistical Analysis

All data were presented as means ± SE. As properly indicated, statistical differences between groups were evaluated using Student’s *t*-test for unpaired samples and, differences among groups were compared by ANOVA followed by the Bonferroni post hoc test to correct for multiple comparisons. Differences were considered statistically significant at *p* < 0.05. All analyses were performed using GraphPad Prism 5.0 (GraphPad Software, San Diego, CA, USA).

## Figures and Tables

**Table 1:** Primers sequences used for semiquantitative PCR.

5-HT2AR	Fw	5′-TCATCATGGCAGTGTCCCTA-3′
	Rv	5′-TGAGGGAGGAAGCTGAAAGA-3′
18s	Fw	5′-GCGCTACACTGACTGGCTC-3′
	Rv	5′-CATCCAATCGGTAGTAGCGAC-3′
